# Intrinsically Stretchable Poly(3,4-ethylenedioxythiophene) Conducting Polymer Film for Flexible Electronics

**DOI:** 10.3390/polym14122340

**Published:** 2022-06-09

**Authors:** Lucija Fiket, Marin Božičević, Lana Brkić, Patricia Žagar, Anamarija Horvat, Zvonimir Katančić

**Affiliations:** Faculty of Chemical Engineering and Technology, University of Zagreb, 10000 Zagreb, Croatia; mbozicevi@fkit.hr (M.B.); lbrkic@fkit.hr (L.B.); pzagar@fkit.hr (P.Ž.); amhorvat@fkit.hr (A.H.)

**Keywords:** conducting polymer, graft copolymer, poly(3,4-ethylenedioxythiophene), acrylate urethane, wearable electronics

## Abstract

The aim of this study was to synthesize an intrinsically stretchable conductive polymer (CP) by atom transfer radical polymerization (ATRP). For this purpose, poly(3,4-ethyilenedioxythiophene) (PEDOT) was synthesized as a backbone, while poly(acrylate-urethane) (PAU) was grafted onto the PEDOT backbone to form graft polymers PEDOT-*g*-PAU. Different concentrations of acrylate-urethane (AU) were used to synthesize PAU side chains of different lengths. The successful synthesis of the obtained intermediates and products (PEDOT-*g*-PAU) was confirmed by infrared spectroscopy and nuclear magnetic resonance. Thermal properties were evaluated by differential scanning calorimetry and thermogravimetric analysis, while conductivity was determined by four-point probe measurement. A simple tensile test was performed to characterize the ductility of the samples. PEDOT-*g*-PAU has shown high stretchability of up to 500% and, therefore, could potentially be used in skin-worn flexible electronics, while additional subsequent doping is required to improve the deterioration of electrical properties after the addition of the insulating urethane layer.

## 1. Introduction

While traditional rigid electronics currently make up the majority of devices used in our everyday lives, the field of flexible electronics has emerged as a viable alternative over the past decade. The growing need for lightweight mobile electronics, real-time health monitoring, and wearable displays has increased the industrial importance of wearable and flexible devices [1,2,3,4]. Skin-inspired wearable devices hold great potential for the next generation of smart wearable electronics. They offer intriguing applications in health monitoring, artificial intelligence, human-machine interfaces, and soft robotics. Despite tremendous research efforts aimed at optimizing wearable devices in terms of flexibility, stretchability, bendability, thickness, and portability, the emerging Internet of Things requires that flexible systems with skin interfaces are equipped with additional features that can mimic skin-like sensing and more [5]. Inorganic materials, such as metals and silica (semiconductors), are highly conductive but have the disadvantage of being rigid and inflexible. The concept of organic conductors/semiconductors has been a hot topic since the discovery of the first conductive polymer (CP) polyacetylene in 1977 [6]. The most obvious advantage of organic electronics over inorganic ones is that they are very flexible and lightweight, which are ideal properties for wearable sensors [7]. CPs have been introduced as one of the classes of organic polymers with metallic or semiconducting properties, such as electrical, magnetic, optical, and electronic properties, while at the same time maintaining the characteristics of conventional organic polymers, such as easier synthesis, lower cost, light weight, and corrosion resistance [8,9,10,11]. These properties have led to an increasing trend of using conducting polymers for wearables [7,12]. These materials can be either insulators or semiconductors in a neutral or undoped form, which can be converted to a doped form by a redox reaction to form delocalized charge carriers. However, an advantage of CPs over many organic polymers is their tunable chemical structure, which can be modified to change the conductivity of the polymer. CPs have charge mobility and π-electron backbones, which are responsible for their low-energy optical transition, better electrical conductivity, and high electron affinity [13]. Doped CPs have electrical conductivities ranging from >1 S cm^−1^ to >1000 S cm^−1^, placing CPs on par with inorganic semiconductors such as silicon and germanium and comparable to conductors such as graphite [14]. The most common form of doping is oxidation, in which anions are inserted into CPs, resulting in positive charge carriers that move through the polymer backbone [15]. Different conductive polymers with long-term electrical and chemical stability have gained popularity in this field, with poly(3,4-ethylenedioxythiophene) (PEDOT) being one of the most important representatives of CPs [9,10]. Due to its unique properties, such as high electrical conductivity, environmental stability, optical transparency in thin films, and processability in aqueous solution [16,17,18], it is used in a wide range of applications, such as organic photovoltaics [19], supercapacitors [20,21], thermoelectric generators [22], transistors [23], sensors [24], and biosensors [25,26]. However, a major drawback of most conductive polymers, and thus PEDOT, is their poor strength and high brittleness, which severely limits their applications. A PEDOT-based copolymer offers a potential solution to this problem and opens the door to a new application of this type of polymers in wearable electronics. The mechanical stretchability of CPs can be improved by incorporating flexible polymer segments into the conjugated backbone [27,28,29]. To improve the flexibility of PEDOT polymer, a graft copolymer was synthesized by grafting the side chains of acrylate urethane (AU) onto the PEDOT backbone by atom transfer radical polymerization (ATRP). ATRP is one of the most powerful and versatile controlled radical polymerization (CRP) processes. It provides precise control over the molecular weight of the polymers, the chain length, and thus the properties of the final product. The polymerization conditions and parameters can be adjusted so that the reaction kinetics can be controlled [30]. AU as soft chains incorporated by grafting onto the rigid PEDOT backbone should make PEDOT flexible by forming non-covalent, hydrogen bonding, crosslinks. Such an approach offers the potential advantage of obtaining self-healable material, since hydrogen bonds can spontaneously reform once broken by stress. When the stress is released, the bonds can be re-formed to restore the original mechanical properties [31]. This is an advantage over the similar literature reports in which PEDOT/polyurethane blends were prepared without hydrogen bonds [32,33]. In this paper, we developed an intrinsically stretchable PEDOT-based graft polymer and to the best of our knowledge, this is the first report on grafting flexible acrylate-urethane side chains on the PEDOT backbone. The synthesis was carried out in four steps and the final product PEDOT-*g*-PAU was obtained and characterized by different techniques. The results confirmed the success of the synthesis of all intermediates, as well as the PEDOT-*g*-PAU product itself.

## 2. Materials and Methods

### 2.1. Materials

Dichloromethane (CH_2_Cl_2_), chloroform (CHCl_3_, 99.94%), tetrahydrofuran (THF) (C_4_H_8_O, 99.94%) and ammonium hydroxide (NH_4_OH, 24.06%) were received from Lach-Ner s.r.o., Neratovice, Czech Republic. α-bromoisobutyryl bromide (C_4_H_6_Br_2_O, >98.0%), 3-thiopheneethanol (C_6_H_8_OS, 98.0%) and N,N,N′,N″,N″-pentamethyldiethylenetriamine (PMDETA) (C_9_H_23_N_3_, >99.0%) were recieved from Tokyo Chemical Industry, Tokyo, Japan. Nitromethane (CH_3_NO_2_, 98+%), copper(I) bromide (CuBr, 98%), dibutyltin dilaurate (DBTDL, [CH_3_(CH_2_)_3_]_2_Sn[O_2_C(CH_2_)_10_CH_3_], iron(III) chloride, anhydrous (FeCl_3_, 98%) and ethyl isocyanate (C_3_H_5_NO, 98%) were received from Alfa Aesar, Haverhill, MA, USA. Triethylamine (C_6_H_15_N, 99%), 3,4-ethylenedioxythiophene (EDOT, 99.0%) silica gel (SiO_2_) and 2-hydroxyethyl acrylate (C_5_H_8_O_3_, 97%) were received from Acros Organics, Geel, Belgium. Magnesium sulfate (MgSO_4_, 99.4%) was received from VWR Chemicals, Leuven, Belgium. Acetone (C_3_H_6_O, 98.0%) and ethanol (C_2_H_5_OH, 96.0%) were received from Gram-mol, Zagreb, Croatia. Methanol (CH_3_OH, 99.9%) was received from Carlo Erba Reagents, Val-de-Reuil, France and diethyl eter (C_4_H_10_O) was received from Macron Fine Chemicals, Radnor, PA, USA. All the solvents were used as received.

### 2.2. Synthesis

#### 2.2.1. Synthesis of Monomer 2-(Thiophene-3-yl)ethyl 2-bromo-2-methylpropanoate (ThBr)

**Scheme 1:**
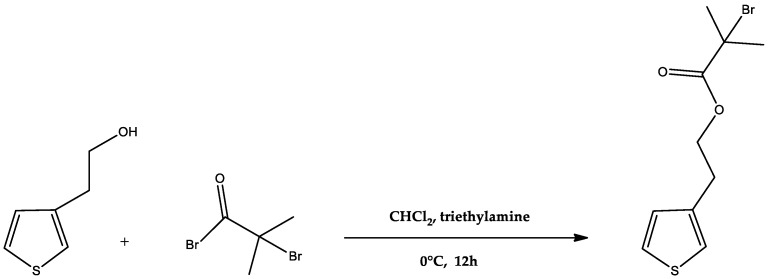
Synthetic Scheme for ThBr monomer.

The first step in the synthesis of PEDOT-*g*-PAU copolymers is the synthesis of monomer 2-(thiophene-3-yl) ethyl 2-bromo-2-methylpropanoate (termed ThBr) according to the literature with modifications [34]. To a 250 mL flask immersed in an ice bath, 100 mL of dichloromethane was added, which was purged with nitrogen for 15 min. Then, 8.7 mL (76.6 mmol, 1 equivalent) of 3-thiophenethanol and 12.2 mL (87.6 mmol, 1.1 equivalent) of triethylamine were added. The reaction mixture was stirred for 10 min to deprotonate 3-thiophenethanol. After 10 min, 10.8 mL of 2-bromoisobutyryl bromide (87.6 mmol, 1.1 equivalent) was added dropwise to the reaction mixture. The reaction mixture was stirred at room temperature for 12 h under a stream of nitrogen to provide an inert atmosphere (Scheme 1). After 12 h, the reaction mixture was washed 3 times with 100 mL of distilled water and once with 100 mL of saturated NaCl solution. The organic layer was separated and dried over MgSO_4_, evaporated on a rotary evaporator, and the residual product was purged with nitrogen. The product was then purified by column chromatography on silica gel using dichloromethane as eluent. The fractions were checked on TLC plates and the desired fractions were collected in a round bottom flask, after which the solvent was evaporated on a rotary evaporator. The product obtained was ThBr monomer. For ThBr, ^1^H NMR (600 MHz, chloroform-d) δ 7.28 (dd, 1H), 7.09–7.04 (m, *J* = 3.0, 1.0 Hz, 1H), 7.00 (dd, *J* = 4.9, 1.3 Hz, 1H), 4.38 (t, 2H), 3.03 (t, *J* = 6.6, 0.7 Hz, 2H), 1.91 (s, 6H).

#### 2.2.2. Synthesis of Poly(3,4-ethylenedioxithiophene-co-2-(thiophene-3-yl) ethyl 2-bromo-2-methylpropanoate) ((poly(EDOT-co-ThBr), termed PEDOT-Br)

The synthesis of the macroinitiators PEDOT-Br was carried out according to the recipe in the literature [35] with modifications, where the monomer ratio was fixed and EDOT:ThBr = 1:1. Masses of 3.000 g (10.8 mmol, 1 equivalent) of ThBr monomer and 1.539 g (10.8 mmol, 1 equivalent) of EDOT monomer were dissolved in 100 mL of chloroform in a reactor that was immersed in an ice bath and purged with nitrogen for 10 min. A total of 5.267 g of (32.5 mmol, 3 equivalents) FeCl_3_ was dissolved in 50 mL of nitromethane, and the prepared solution was transferred to a dropping funnel. The oxidant solution was added dropwise to the reaction mixture at 0 °C under a stream of nitrogen. After the reaction had been carried out for 30 min under a stream of nitrogen, the nitrogen was replaced by argon. The reaction was carried out for 24 h and then quenched by adding 200 mL of methanol. The product obtained is a copolymer of PEDOT-Br in the doped state, which was filtered and added to 200 mL of a solution of NH_4_OH:CH_3_OH = 1:1 to achieve dedoping and stirred in this solution for 1 h. The dedoped polymer was then filtered and washed with methanol to remove the residual FeCl_3_. The product obtained consisted of soluble and insoluble PEDOT-Br, and to isolate the soluble part, Soxhlet extraction was performed using chloroform as solvent. A solution of the polymer in chloroform was obtained, after which the solvent was evaporated in a rotary evaporator. Drying yielded 3.2% of a black powder of the macroinitiator PEDOT-Br.

#### 2.2.3. Synthesis of Acrylate Urethane (AU) Monomer

**Scheme 2:**
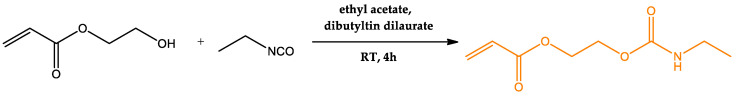
Synthetic Scheme for AU monomer.

The synthesis of the acrylate-urethane monomer was carried out according to the recipe in the literature [36] with modifications (Scheme 2). A total of 4 mL (54.6 mmol, 1 equivalent) of ethyl isocyanate and 5.9 mL (49.5 mmol, 1.1 equivalents) of 2-hydroxyethyl acrylate were added to the reactor with constant stirring. Then, 5 drops of dibutiline dilaurate were added to the reaction mixture. The synthesis was carried out under a stream of nitrogen with constant stirring for 4 h. It was then diluted with 20 mL of dichloromethane and washed with deionized water to remove the unreacted 2-hydroxyethyl acrylate. The remaining deionized water was removed by adding Na_2_SO_4_, after which the mixture was filtered. The urethane acrylate obtained was characterized by infrared spectroscopy with Fourier transform and nuclear magnetic resonance. For AU, ^1^H NMR (300 MHz, chloroform-d) δ 6.44 (dd, *J* = 17.3, 1.5 Hz, 1H), 6.15 (dd, *J* = 17.3, 10.4 Hz, 1H), 5.86 (dd, *J* = 10.4, 1.5 Hz, 1H), 4.73 (s, 1H), 4.45–4.25 (m, 4H), 3.23 (p, *J* = 6.9 Hz, 2H), 1.14 (t, *J* = 7.3 Hz, 3H). 

#### 2.2.4. Graft Copolymerization of Poly(3,4-ethylenedioxithiophene-co-2-(thiophene-3-yl) ethyl 2-bromo-2-methylpropanoate)-graft-poly(acrylate-urethane) ((PEDOT-Br-*g*-PAU), termed PEDOT-*g*-PAU)

**Scheme 3:**
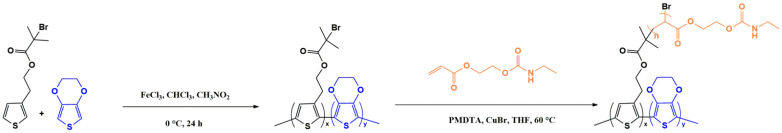
Synthetic scheme for PEDOT-*g*-PAU graft copolymer.

Four ATRP reactions with different AU monomer ratios were carried out under identical ATRP conditions to obtain graft copolymers with different compositions and to investigate the influence of AU content on the properties of the final product. PEDOT-*g*-PAU graft copolymers were prepared in the following ratios of the ATRP initiator: AU = 1:30, 1:50, 1:100 and 1:150. PEDOT-*g*-PAU products named PEDOT-*g*-PAU 30, PEDOT-*g*-PAU 50, PEDOT-*g*-PAU 100, and PEDOT-*g*-PAU 150 were obtained. The synthesis of PEDOT-*g*-PAU was prepared using a conventional atom transfer radical polymerization ATRP technique following the recipes from the literature with modifications (Scheme 3) [34,37]. The molar ratio of ATRP macroinitiator:CuBr:PMDETA was 1:2.2:4.4 in all reactions. A total of 20 mL of THF was purified with nitrogen for 20 min, then PEDOT-Br, AU, and PMDETA were added. To a 100 mL flask, CuBr was added, which was also kept under nitrogen atmosphere. The solution was then transferred to a CuBr flask and stirred at 60 °C on a magnetic stirrer under argon for 4 h. After 4 h, the reaction was quenched by exposing the reaction mixture to air. After cooling, the mixture was diluted with 85 mL of THF and centrifuged (*T* = 20 °C, 4500× *g* rpm) for 1 h with precipitation of the copper complexes. The solution was then decanted and most of the solvent was removed using a rotary evaporator. The polymer was then precipitated in 200 mL of diethyl ether, the precipitate was collected by decantation and suspended in fresh 200 mL of diethyl ether for 24 h. The precipitate was then removed by a rotary evaporator. The resulting precipitate was collected by centrifugation and dried for 12 h in vacuum.

### 2.3. Characterizations and Instruments

#### 2.3.1. Fourier-Transform Infrared Spectroscopy (FTIR)

The synthesized samples were characterized by Fourier-transform infrared spectroscopy (FTIR). Measurements were performed with a PerkinElmer Spectrum One spectrophotometer (PerkinElmer, Waltham, MA, USA) using an attenuated total reflectance (ATR) chamber with a ZnSe crystal in the measurement range 4000−50 cm^−1^. The samples were used in their original powder form without prior preparation.

#### 2.3.2. Nuclear Magnetic Resonances (NMR)

^1^H spectra were recorded using a Bruker Avance III HD spectrometer (400 MHz) (Bruker, Billerica, MA, USA). Chemical shifts (*δ*) were expressed in parts per million (ppm) and referenced to tetramethylsilane (TMS) in deuterated chloroform (CDCl_3_) at room temperature.

#### 2.3.3. Gel Permeation Chromatography (GPC)

PEDOT-Br and PEDOT-*g*-PAU samples were prepared by dissolving ~5 mg of the samples in 375 mg of tetrahydrofuran (THF) (1:75 ratio) and injected into the device. The injection volume of the sample was approximately 300 μL. Measurements were performed using a PL-GPC 20 PolymerLaboratories chromatography instrument (PolymerLaboratories, Shropshire, UK) equipped with a refractometric sensor. The separation unit consists of two PLgelMixed-B columns connected in series and filled with poly(styrene/divinylbenzene) terpolymer gel with a particle size 3–100 μm and THF as solvent.

#### 2.3.4. Differential Scanning Calorimetry (DSC)

The glass transition temperature (*T*_g_) of the synthesized samples was determined by differential scanning calorimetry (DSC) on a Mettler Toledo DSC 823e calorimeter (Mettler Toledo, Columbus, OH, USA). The samples were tested by heating and cooling in a nitrogen atmosphere at a flow rate of 50 mL min^−1^. The mass of each sample was approximately 5–10 mg. Thermograms were recorded in the following two cycles: heating from −20 °C to 130 °C at 10 °C min^−1^; isothermal for 2 min at 130 °C; cooling to −20 °C at 10 °C min^−1^; isothermal for 2 min at −20 °C; heating from −20 to 130 °C at 10 °C min^−1^.

#### 2.3.5. Thermogravimetric Analysis (TGA)

Thermogravimetric analysis was performed to determine the thermal stability of the test sample using the TA Instruments Q500 (TA Instruments, New Castle, DE, USA). Measurements were performed under an inert nitrogen atmosphere at a flow rate of 60 mL min^−1^. The mass of the sample was ~5 mg, and the heating rate was 10 °C min^−1^ in the measurement range from 25 °C to 700 °C. The onset of decomposition, *T*_90_, temperature at which 50% of the sample weight remains, *T*_50_, and char residues were determined for the samples. Three replicates were run for each sample and the average value was reported. Uncertainty of *T*_90_ and *T*_50_ was less than 1.9 °C, while char residue uncertainty was 1.4 mass% (2σ).

#### 2.3.6. Four-Point Probe Method (4PP)

Electrical conductivity (*σ*) was determined by measuring the electrical resistance (*R*) of the samples with a Keysight 34,461 61/2 Digit Multimeter instrument (Keysight, Santa Rosa, CA, USA) on a smooth sample surface using a 4-point probe method. The distance between the probes was 1.6 mm. The PEDOT-Br sample was prepared in the form of a pastille, the thickness of which was measured with a caliper and was 145 μm. The conductivity of PEDOT-*g*-PAU graft copolymers was measured on films prepared for tensile test (described in the Section 2.3.7) and their thickness was 8 to 10 μm. Based on the measured resistance and the thickness of the pastille and the PEDOT-*g*-PAU films, the electrical resistance was calculated according to the following equation [38]:

(1) ρ=πdR/ln2 (Ω m)

where *R*—electrical resistance, *ρ*—electrical resistance, *d*—thickness of the sample.

The reciprocal of the electrical resistance is equal to the electrical conductivity.

(2) $$\sigma =1/\rho \;(S\;m^{-1})$$

The conductivity value was the average of the measurement at 10 to 15 different locations on the pastille and graft copolymer films.

#### 2.3.7. Tensile Test

A tensile test of the synthesized graft copolymer PEDOT-*g*-PAU was carried out to determine the stretchability of the obtained product in order to determine the possibility of using this material in the flexible electronics. The tests were carried out by dissolving the samples in THF and applying the obtained polymer solution to a low-density polyethylene (LDPE) film (dimensions 50 mm × 15 mm × 60 µm). Solution was applied by universal applicator where the thickness was set to 60 µm. The final thicknesses of the graft copolymer films after THF evaporation were 8–10 µm. LDPE substrate was then manually stretched until it cracked.

## 3. Results and Discussion

### 3.1. Fourier-Transform Infrared Spectroscopy (FTIR)

**Figure 1:**
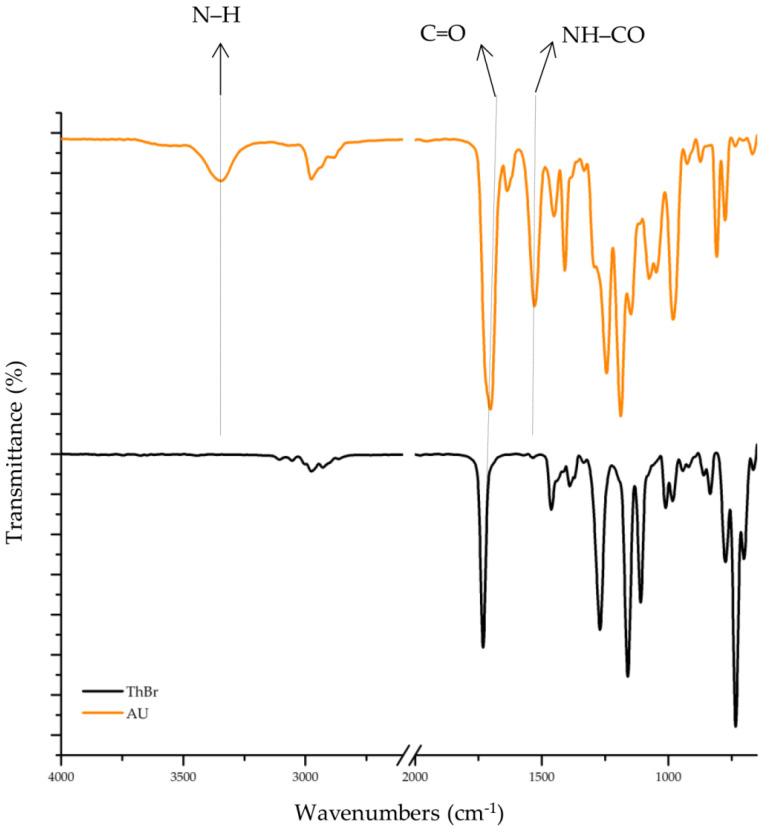
FTIR spectra of monomers ThBr and acrylate-urethane.

**Figure 2:**
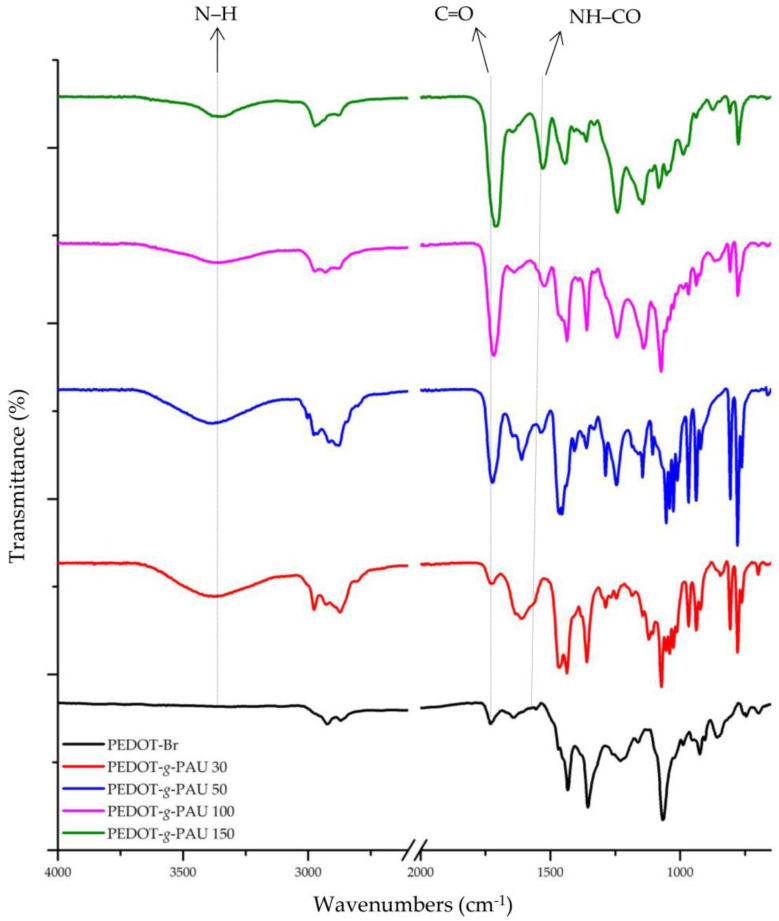
FTIR spectra of PEDOT-Br macroinitiator and PEDOT-*g*-PAU copolymers.

In order to identify the functional groups of the synthesized products and determine the success of the synthesis, the samples were analyzed by FTIR spectroscopy and Table 1 shows all the signals for the characteristic group of all the products. The FTIR spectra of the ThBr and acrylate-urethane monomers are shown in Figure 1. They all show the characteristic signals indicating that the desired monomers were successfully synthesized. Bands with peaks at 2980 cm^−1^ and 2860 cm^−1^ were attributed to the stretching of C–H bonds of the methyl groups, and bands with peaks at 1730 cm^−1^ and 1703 cm^−1^ indicate the presence of a carbonyl group in the ThBr and AU monomers, respectively. Characteristic bands indicating the stretching of the C=C and C–C bonds of the thiophene ring in the ThBr monomer are visible in the range from 1462 cm^−1^ to 1389 cm^−1^. Figure 2 shows the spectra of the PEDOT-Br macroinitiators and the main products of the PEDOT-*g*-PAU copolymer obtained by grafting AU onto PEDOT-Br macroinitiators, in which the signals of all characteristic functional groups can also be observed. Comparing the spectra of the PEDOT-Br macroinitiators and the PEDOT-*g*-PAU graft copolymers, the appearance of characteristic signals for N–H bonds, NH–CO bonds and C–N bonds can be observed. The observed broad band in the range from 3381 cm^−1^ to 3360 cm^−1^ corresponds to N–H stretching, while the appearance of signals in the spectra of the graft copolymers in the range from 1564 cm^−1^ to 1521 cm^−1^ and at about 1245 cm^−1^ is due to NH–CO and C–N bonds, respectively. It should be mentioned that O–H stretching from humidity could contribute to some extent to the broad peak in the 3600–3200 cm^−1^ range. No water was used in the ATRP reaction and the samples were dried in a vacuum dryer, but it cannot be excluded that the samples adsorbed a small amount of moisture from the air. However, we assume that it is a small amount that could have only a minor effect on the IR spectra. The appearance of the new bands is the result of grafting AU onto the macroinitiator PEDOT-Br, in whose spectrum these bands are not present, indicating the success of the synthesis. It is important to note that the signal intensity of the carbonyl group C=O increases with the increase in the content of acrylate-urethane monomers in the grafted copolymers.

### 3.2. Nuclear Magnetic Resonances (NMR)

NMR analysis was performed to gain insight into the structure of the synthesized products and to determine the success of the synthesis of ThBr, PEDOT-Br, AU, and the final product PEDOT-*g*-PAU.

**Figure 3:**
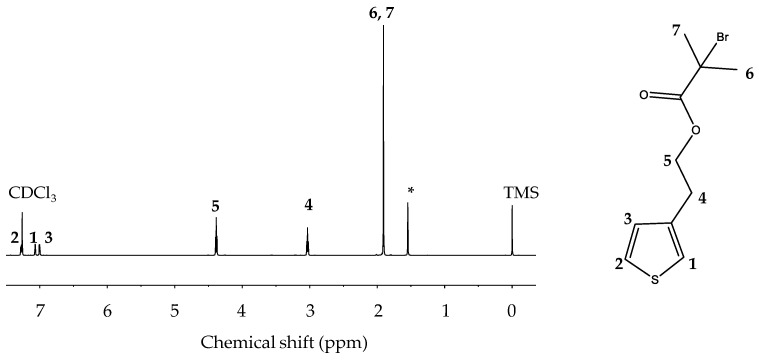
^1^H NMR spectrum of ThBr monomer.

Figure 3 shows the ^1^H NMR spectrum of the synthesized ThBr monomer and confirms the success of the synthesis. At 1.91 ppm, a singlet assigned to the protons of two symmetric CH_3_ groups at positions 6 and 7 of the ThBr monomer is visible. In the range from 3 ppm to 4.38 ppm, two triplets assigned to the protons of CH_2_ groups are observed. The triplet at 4.38 ppm corresponds to the protons at position 5 of the ThBr and the triplet at 3.03 ppm corresponds to the protons at position 4. The doublet of the doublet at 7.28 ppm corresponds to the proton at position 2 of the thiophene ring. At a shift of 7.0 ppm, there is a doublet of the doublet corresponding to the proton at position 3, while the multiplet at 7.07 ppm corresponds to the proton at position 1 of the thiophene ring. Two singlet values at 1.54 ppm (marked with *) and 7.27 ppm related to the solvents used can also be observed in the spectrum, including water and chloroform, respectively.

**Figure 4:**
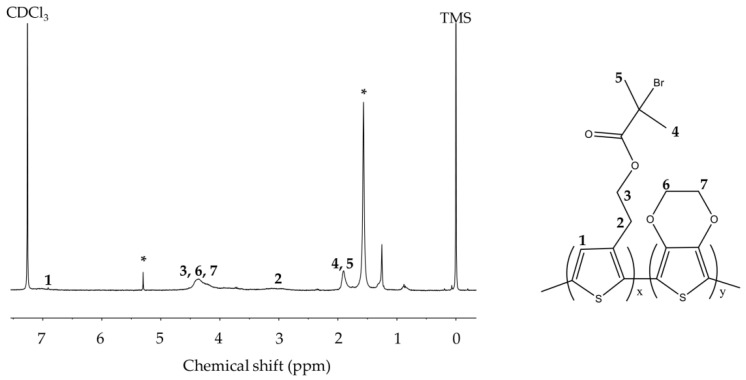
^1^H NMR spectrum of PEDOT-Br macroinitiator.

Figure 4 shows the ^1^H NMR spectra of PEDOT-Br. At 1.91 ppm, a signal characteristic of ThBr is observed that corresponds to the protons of two equivalent methyl groups. At 4.36 ppm, the signal generated by the overlap of the signals characteristic of the protons of the CH_2_ group of the ThBr and the signal corresponding to the EDOT monomer is observed (positions 3, 6 and 7). At 6.92 ppm, a signal corresponding to the protons of the thiophene ring (position 1) is observed. The absence of two signals visible in the spectrum of ThBr monomers in this part of the spectrum confirms the success of macroinitiator synthesis. Signals related to residual solvents can also be observed in the spectrum. A singlet bound to water is visible at 1.57 ppm, a singlet bound to dichloromethane at 5.30 ppm (both marked with *), and a singlet bound to chloroform at 7.26 ppm.

**Figure 5:**
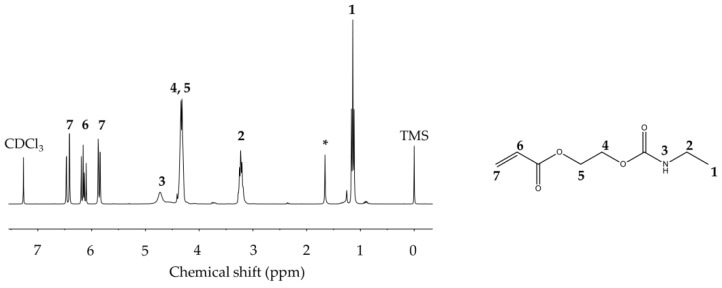
^1^H NMR spectrum of AU monomer.

The NMR spectrum of the AU monomer is shown in Figure 5. At 1.14 ppm, a triplet is present relative to the protons of the methyl group at position 1 of the AU. The signal at 3.23 ppm relates to the protons of the CH_2_ group at position 2 of the AU, which are shaded due to the close proximity of the N atom, while in the range from 4.55 ppm to 4.25 ppm, a multiplet is visible corresponding to the protons at positions 4 and 5 of the AU. The singlet at 4.73 ppm corresponds to the proton of the NH group, while the triplet at 6.15 ppm refers to the proton at position 6. The doublets at 5.86 ppm and 6.44 ppm refer to the 2 protons at position 7 of the AU. Since rotation is disabled by the presence of a double bond, these two protons are not equivalent and signal splitting occurs. The spectrum also shows singlets at 7.27 ppm and 1.66 ppm (marked with *), indicating residual chloroform and water, respectively.

**Figure 6:**
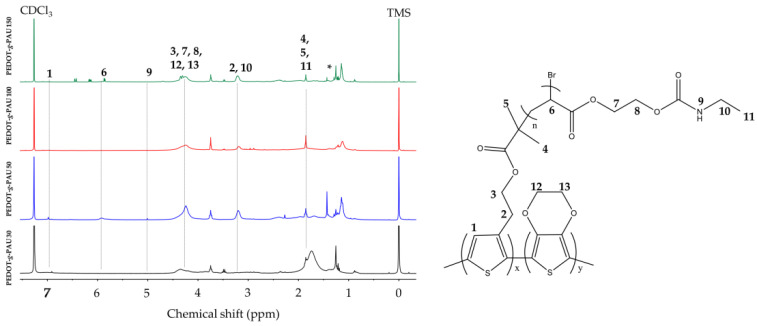
^1^H NMR spectrum of graft copolymer PEDOT-*g*-PAU.

Figure 6 shows the ^1^H NMR spectra of the PEDOT-*g*-PAU graft copolymer. At 3.20 ppm, 5.92 ppm and 6.52 ppm signals corresponding to protons of the AU can be observed, while at 5.01 ppm a signal corresponding to the NH group can be observed. At about 7 ppm, signals associated with protons of the thiophene ring can be observed, and at 4.24 ppm, a broad signal corresponding to protons at positions 2, 3, 4, 8, and 9 can be observed. Since the doublets at 5.86 ppm and 6.44 ppm that refer to the two protons at position 7 of the AU are no longer present (Figure 5), it can be concluded that polymerization has occurred and that it is not just a mixture of the PEDOT-Br macroinitiator and AU monomer.

### 3.3. Gel Permeation Chromatography (GPC)

The values of numerical (*M*_n_) and mass average (*M*_w_) molecular weights and dispersity (*Đ*) for the obtained products PEDOT-Br and PEDOT-*g*-PAU (Table 2) were obtained by the size exclusion method.

The average molecular weight values for the macroinitiator PEDOT-Br are higher than the literature values [37], and from the obtained results, the synthesized graft copolymers PEDOT-*g*-PAU have higher average molecular weight values compared to PEDOT-Br. Such a result is expected, since grafting the side chains AU onto PEDOT-Br should increase the molecular weights. With the increasing AU content in the copolymers, a gradual increase in the numerical and mass-average molecular weight is observed. *M*_n_ and *M*_w_ increased more than double for sample PEDOT-*g*-PAU 150 when compared to the macroinitiator. The ratio of the numerical and mass-average molecular weights was used to determine dispersion, which is a measure of the heterogeneity of the system. The relatively low *Đ* values of copolymers indicate a narrower molecular weight distribution when compared to PEDOT-Br. *Đ* in the range of 1.21 to 1.36 for graft copolymers PEDOT-*g*-PAU indicates the narrow distribution of molecular weight, which can be expected from controlled radical polymerizations such as ATRP [40]. Much wider distribution with the dispersity of 1.53 is visible for PEDOT-Br macroinitiator where oxidative polymerization was used.

### 3.4. Differential Scanning Calorimetry (DSC)

**Figure 7:**
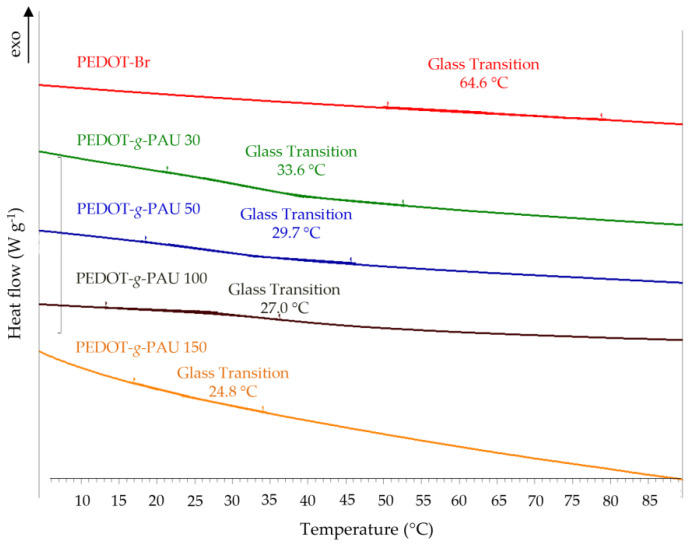
DSC curves of the macroinitiator PEDOT-Br and the obtained graft copolymers PEDOT-*g*-PAU.

DSC analysis was performed on samples of PEDOT-Br macroinitiator and grafted PEDOT-*g*-PAU copolymers to determine the glass transition temperature of the samples (Figure 7). As can be observed in the figure, the very weak signal of *T*g of PEDOT-Br is visible at 64.6 °C and it corresponds to the literature [37]. By introducing soft AU side chains, the graft copolymers show a significant decrease in the glass transition temperature to 33.6 °C, 29.7 °C, 27.0 °C and 24.8 °C for PEDOT-*g*-PAU 30, PEDOT-*g*-PAU 50, PEDOT-*g*-PAU 100, and PEDOT-*g*-PAU 150, respectively, reflecting their much softer nature than the PEDOT-Br backbone. The introduction of AU side chains into the structure of the PEDOT-Br copolymer resulted in the formation of an amorphous phase within the polymer structure, which increased its flexibility. The obtained PEDOT-*g*-PAUs exhibited a glass transition below body temperature, which enables their potential application in wearable electronics.

### 3.5. Thermogravimetric Analysis (TGA)

**Figure 8:**
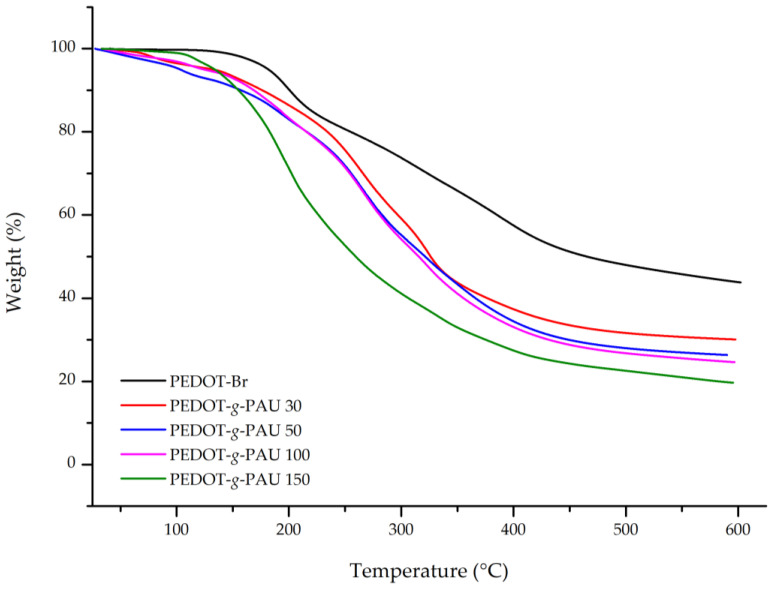
TG curves of PEDOT-Br macroinitiator and PEDOT-*g*-PAU graft copolymer.

The thermal properties of PEDOT-Br macroinitiator and PEDOT-*g*-PAU graft copolymers were studied by TG analysis. The obtained TG curves showing the dependence of mass loss on temperature are shown in Figure 8. From the obtained thermograms, the temperature at 10% mass loss, *T*_90_, the temperature at 50% mass loss, *T*_50_, and the residual mass at 600 °C after pyrolytic degradation in N_2_ are evident (see Table 3). The residual mass after pyrolytic degradation indicates the mass of the sample, which consists of charred structures formed as a product of polymer degradation. From the TG curves, it is clear that the degradation of all the samples occurs in several steps. The first mass loss at temperatures up to 100 °C refers to residual solvents, followed by the decomposition of the remaining monomers in the sample. At higher temperatures, the resulting products of PEDOT-Br and PEDOT-*g*-PAU decompose. The results of the thermal analysis of the PEDOT-Br macroinitiators show the temperatures of the onset of decomposition above 200 °C, the *T*_50_ temperature of 464.9 °C and the residue after pyrolysis of 41.4%, indicating the good thermal stability of this polymer. The obtained graft copolymers PEDOT-*g*-PAU have lower *T*_90_ and *T*_50_ values compared to PEDOT-Br. The *T*_90_ value correctly decreases from 201 °C to 153 °C with increasing PAU content, while the *T*_50_ value also correctly decreases from 465 °C as for the macroinitiators to 260 °C as for PEDOT-*g*-PAU. The same is true for the char residue, which ranges from 19% to 29% for grafted polymers. Therefore, it can be concluded that the introduction of AU side chains decreases the thermal stability of the polymer, which was expected since it is known from the literature that urethane bonds begin to dissociate at around 150 °C [41].

### 3.6. Four-Point Probe Method (4PP)

The electrochemical properties of the synthesized PEDOT-Br macroinitiator and the obtained PEDOT-*g*-PAU graft copolymer were tested by the four-point probe method. The conductivity of electrically conductive polymers largely depends on the state of doping. The macroinitiator PEDOT-Br exhibited a conductivity of 495.7 ± 2.1 S m^−1^. The conductivity of PEDOT-*g*-PAU decreased due to the insulating PAU side chains. The determined conductivities of PEDOT-*g*-PAU were 377.9 ± 3.4 S m^−1^, 302.1 ± 1.1 S m^−1^, 238.0 ± 3.2 S m^−1^ and 53.3 ± 2.1 S m^−1^ for the copolymers PEDOT-*g*-PAU 30, PEDOT-*g*-PAU 50, PEDOT-*g*-PAU 100 and PEDOT-*g*-PAU 150, respectively. Comparing the electrical conductivity of PEDOT-Br macroinitiators and PEDOT-*g*-PAU grafted copolymers, we find that the electrical conductivity decreases with increasing AU content in the grafted copolymers, which is expected since AU are non-conductive and decrease the electrical conductivity. Nonetheless, although conductivity was relatively low, some of the electroactivity was preserved, which could potentially be enhanced by further doping procedures. Considering the absence of any kind of dopant, the conductivities are not so low compared to the similar systems found in the literature. Ding et al. [32] obtained conductivities in the range of 30 S m^−1^ to 200 S m^−1^, while Taroni et al. [33] obtained very high results of 7900 S m^−1^. In both cases, PEDOT was doped with poly(styrenesulfonate) (PSS), so it can be assumed that higher conductivities are possible with subsequent doping.

### 3.7. Tensile Test

**Figure 9:**
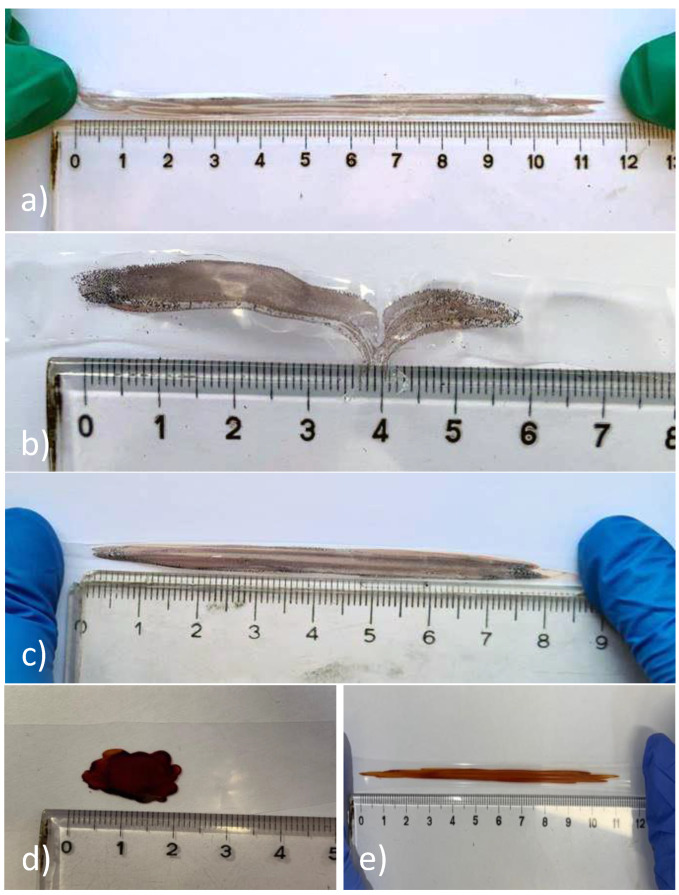
Stretching of the final product, (**a**) PEDOT-*g*-PAU 30, (**b**) PEDOT-*g*-PAU 50, (**c**) PEDOT-*g*-PAU 100, (**d**) PEDOT-*g*-PAU 150 before stretching; (**e**) PEDOT-*g*-PAU 150 stretched at cca 500%.

**Figure 10:**
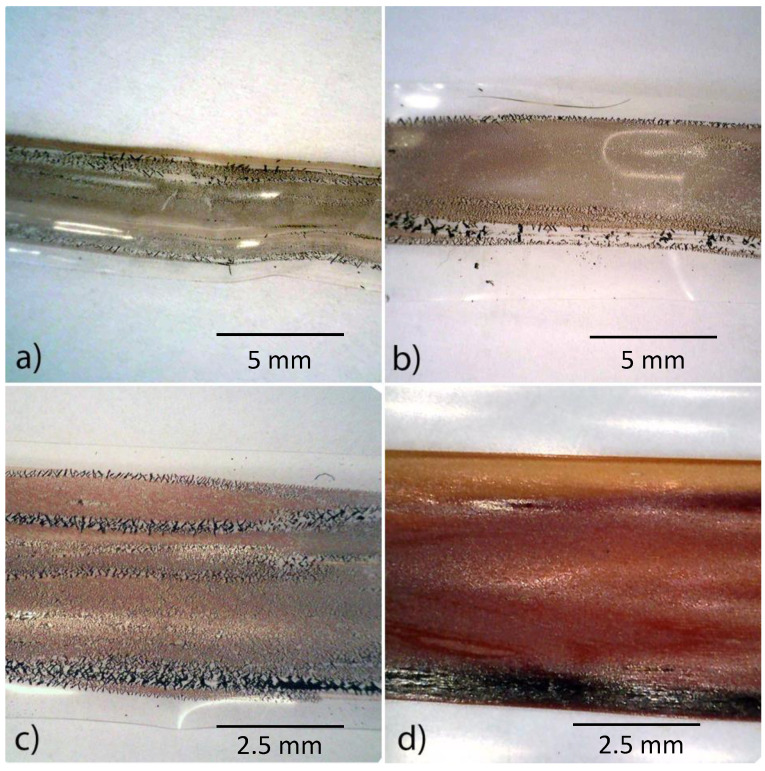
Stretched samples at higher magnification, (**a**) PEDOT-*g*-PAU 30, (**b**) PEDOT-*g*-PAU 50, (**c**) PEDOT-*g*-PAU 100; (**d**) PEDOT-*g*-PAU 150.

To test the stretchability of the synthesized samples, a graft copolymer THF solution was manually applied in a thin layer to a low-density polyethylene film. After solvent evaporation, the films were stretched by hand, and the appearance of the PEDOT-*g*-PAU films was observed. Pictures of films are shown in Figure 9. All films were able to stretch to about 500% of their starting length, which was approximately 2 cm. At those extensions, LDPE substrates started to break, so it can be assumed that the synthesized copolymers could be stretched even higher. All samples were able to stretch to 500% with the exception of sample PEDOT-*g*-PAU 50, where the LDPE substrate broke at around 300% (Figure 9b). A digital microscope was used to closer observe the appearance of films in stretched condition at around 300% and the pictures are shown in Figure 10. It can be observed that the samples PEDOT-*g*-PAU 30 and PEDOT-*g*-PAU 50 started to peel and delaminate from the substrates, indicating a lack of adhesion as they neared the elongation limit. Somewhat better behviour was observed for sample PEDOT-*g*-PAU 100 where delamination started to occur only where the layer of applied polymer was thicker. Finally, the sample PEDOT-*g*-PAU 150 with the highest amount of PAU showed that stretched layer is stable, without any traces of peeling from LDPE. Even the edges (Figure 10d, lower edge) where the applied layer was thicker wereable to fully withstand substrate stretching.

## 4. Conclusions

Obtaining highly stretchable yet highly conductive organic material for flexible electronics is still a major challenge. In this paper, a new PEDOT-based polymer, grafted with poly(acrylate-urethane) side chains, is presented. The successful synthesis of graft copolymers with different side chain lengths was confirmed by FTIR and NMR analyses. The incorporation of soft urethane branches significantly decreased the stiffness of PEDOT, as evidenced by a reduction in the glass transition temperature by 30 to 40 °C compared to PEDOT macroinitiator. The obtained graft copolymers have a glass transition lower than body temperature, which allows their use in flexible skin-mounted electronics. The grafted polymers also exhibited high tensile properties, where elongation of up to 500% was achieved when casted on LDPE substrate. The electrochemical properties of the synthesized PEDOT-*g*-PAU samples were reduced by the addition of insulating urethane side branches, but could be improved by the subsequent implementation of a doping process.

## Figures and Tables

**Table 1:** Relevant FTIR frequencies (cm^−1^) for monomers and polymers [34,39].

Assignment (cm^−1^)	NH	C–H	C=O	NH–CO	C–C, C=C Thiophene Ring	C–N	C–O	C–S
ThBr	−	2975, 2864	1732	−	1462, 1389	−	1160	983
PEDOT-Br	−	2924, 2869	1730	−	1433, 1355	−	1163	988
AU	3346	2976, 2882	1703	1528	−	1243	1187	−
PEDOT-*g*-PAU 30	3374	2977, 2870	1726	1564	1436, 1360	1246	1145	988
PEDOT-*g*-PAU 50	3381	2971, 2875	1723	1533	1464, 1361	1245	1145	986
PEDOT-*g*-PAU 100	3360	2975, 2872	1716	1521	1436, 1360	1243	1140	985
PEDOT-*g*-PAU 150	3360	2972, 2876	1709	1529	1442, 1362	1241	1144	988

**Table 2:** Values of numerical (*M*_n_), mass (*M*_w_) average molecular weights and dispersity (*Đ*).

Sample	*M*_n_	*M*_w_	*Đ*
PEDOT-Br	84 477 ± 1151	129 250 ± 3484	1.53 ± 0.03
PEDOT-*g*-PAU 30	116 434 ± 1541	140 885 ± 983	1.21 ± 0.01
PEDOT-*g*-PAU 50	131 770 ± 2606	162 077 ± 6120	1.23 ± 0.03
PEDOT-*g*-PAU 100	175 758 ± 3740	227 020 ± 5941	1.29 ± 0.04
PEDOT-*g*-PAU 150	193 655 ± 5477	263 370 ± 6178	1.36 ± 0.01

**Table 3:** Thermal properties of PEDOT-Br macroinitiators and copolymers PEDOT-*g*-PAU.

Sample	*T*_90_ (°C)	*T*_50_ (°C)	Residue (%)
PEDOT-Br	200.7	464.9	41.4
PEDOT-*g*-PAU 30	176.3	326.3	29.0
PEDOT-*g*-PAU 50	157.2	320.8	25.2
PEDOT-*g*-PAU 100	168.9	315.3	23.3
PEDOT-*g*-PAU 150	153.0	259.6	19.0

## Data Availability

The data presented in this study are available on request from the corresponding author.
